# The Impact of Alcoholic Beverages on Human Health

**DOI:** 10.3390/nu13124417

**Published:** 2021-12-10

**Authors:** Peter Anderson

**Affiliations:** 1 Population Health Sciences Institute, Newcastle University, Baddiley-Clark Building, Newcastle upon Tyne NE2 4AX, UK; peteranderson.mail@gmail.com; 2 Department of Health Promotion, CAPHRI Care and Public Health Research Institute, Maastricht University, 6211 LK Maastricht, The Netherlands

As summarized in the World Health Organization’s latest Global Status Report on Alcohol, the pleasure of alcohol is indicated by the fact that, worldwide, just over two-fifths of the population aged 15+ years drink alcohol; 2.3 billion people, consuming nearly 35 billion litres of pure ethanol a year, equivalent to just over three drinks (33 g of pure ethanol) a day (see [1]).

The pain of alcohol is indicated in the collection of papers in this special issue: ethanol in alcoholic beverages is toxic to human health, causing 7.1% of all deaths amongst those aged less than 70 years (two million deaths a year), with the three top causes of death being cirrhosis of the liver, road injuries, and tuberculosis [1]. In addition, through a combination of brain damage due to consuming alcohol and genetic predisposition, some 4% of adults experience what is termed alcohol dependence, a complex behavioural syndrome that has at its core the inability to control alcohol consumption despite adverse social, occupational or health consequences [2].

Ethanol:

- Is a teratogen [3];
- Is genotoxic and a carcinogen [4,5];
- Is hepatotoxic [6];
- Is neurotoxic to the brain [2];
- Causes injuries [7];
- Causes a range of cardiovascular diseases [8];
- Increases the risk of a range of communicable diseases, including HIV, TB, pneumonia, and COVID-19 infection [9]; and
- May or may not increase the risk of overweight and obesity [10].

For most conditions, the dose–response curves increase from zero consumption upwards, with many curves being exponential [11]. The exception to this is that some alcohol consumption protects some people against ischaemic diseases to some degree, with potential benefits occurring at about 1 drink every other day [8]; this is an hormetic effect to an environmental agent characterized by a low dose beneficial effect and a high dose toxic effect, that, for ethanol, may be a consequence of human ancestral exposure to naturally occurring low levels of ethanol from ripe fruit [12]. On the other side, though, margins of exposure analysis indicate that the present average daily consumption amongst drinkers across the world (33 g of ethanol a day) exceeds typically accepted thresholds indicating health risks for carcinogens by some 10,000 [5].

The question this supplement raises, is the pleasure worth the pain?

Governments and governmental organizations seem to consider that the pleasure is worth the pain. As Stockwell and colleagues point out, it is possible to provide very specific and detailed advice to governments regarding the public health consequences of policy decisions in such concrete terms as how many people will become ill, injured or die prematurely from alcohol-related reasons if policy X or Y is not introduced [13]. Yet, almost all countries fall far short of implementing effective public health policies to reduce the harm done by alcohol [13]. Further, despite alcohol being a carcinogen, [4,5], at least in Europe, health warning labels are notable by their absence [14]. Additionally, even for people who run into problems, including alcohol dependence, there seems a lack of care and treatment. As Nutt and colleagues point out, it remains the case that, to date, only three pharmacotherapies are licensed for alcohol dependence and only 9% of such individuals receive such treatment [2]; there is simply no moral outrage from non-governmental organizations.

The alcohol industry also seems to consider that the pleasure is worth the pain. Product reformulation of existing products to contain less alcohol, and more extensive market penetration of no and low alcohol products could lead to consumers drinking less ethanol (see [15]). Yet, overall, there seem to be only very limited moves in this direction [15], and the alcohol industry continues to counter the implementation of effective policies that could reduce the harm done by alcohol [13].

Why is there a dissonance between what the science says about alcohol’s toxicity, and the failure to prevent two million deaths a year amongst the under seventies and provide adequate treatment to the 4% of adults who experience alcohol dependence? Stockwell et al. [13] mention four reasons:

1. lack of public awareness of both need and the effectiveness of policies;
2. lack of government regulatory mechanisms to implement effective policies;
3. alcohol industry lobbying; and,
4. a failure from the public health community to promote specific and feasible actions as opposed to general principles, e.g., ‘increased prices’ or ‘reduced affordability’.

What would be a litmus test of change that we take the pain of alcohol seriously?

The simple specific take of this editorial:

The alcohol industry places a warning label on its products: **ALCOHOL CAUSES CANCER**.

Such an (unlikely) action would:

1. Shame governments for failing to protect their citizens against a known carcinogen; and,
2. Demonstrate that the alcohol industry is serious in taking responsibility for its products.
